# Tuning of strain and surface roughness of porous silicon layers for higher-quality seeds for epitaxial growth

**DOI:** 10.1186/1556-276X-9-348

**Published:** 2014-07-11

**Authors:** Marwa Karim, Roberto Martini, Hariharsudan Sivaramakrishnan Radhakrishnan, Kris van Nieuwenhuysen, Valerie Depauw, Wedgan Ramadan, Ivan Gordon, Jef Poortmans

**Affiliations:** 1 KACST-Intel Consortium Center of Excellence in Nano-manufacturing Applications (CENA), Riyadh 11442, KSA; 2 Interuniversity Microelectronics Center (IMEC), Kapeldreef 75, Leuven 3001, Belgium; 3 Physics Department, Faculty of Science, Alexandria University, Alexandria 21511, Egypt; 4 Department of Electrical Engineering, KU Leuven, Leuven 3000, Belgium; 5 UHasselt, Martelarenlaan 42, Hasselt 3500, Belgium

**Keywords:** Porous silicon, Strain, Surface roughness, Epitaxial growth, Layer-transfer process, Annealing time, Low-porosity layer, Seed layer, High-porosity layer

## Abstract

Sintered porous silicon is a well-known seed for homo-epitaxy that enables fabricating transferrable monocrystalline foils. The crystalline quality of these foils depends on the surface roughness and the strain of this porous seed, which should both be minimized. In order to provide guidelines for an optimum foil growth, we present a systematic investigation of the impact of the thickness of this seed and of its sintering time prior to epitaxial growth on strain and surface roughness. Strain and surface roughness were monitored in monolayers and double layers with different porosities as a function of seed thickness and of sintering time by high-resolution X-ray diffraction and profilometry, respectively. Unexpectedly, we found that strain in double and monolayers evolves in opposite ways with respect to layer thickness. This suggests that an interaction between layers in multiple stacks is to be considered. We also found that if higher seed thickness and longer annealing time are to be preferred to minimize the strain in double layers, the opposite is required to achieve smoother layers. The impact of these two parameters may be explained by considering the morphological evolution of the pores upon sintering and, in particular, the disappearance of interconnections between the porous seed and the bulk as well as the enlargement of pores near the surface. An optimum epitaxial growth hence calls for a trade-off in seed thickness and annealing time, between minimum-strained layers and rougher surfaces.

**PACS codes:**

81.40.-z Treatment of materials and its effects on microstructure, nanostructure, and properties; 81.05.Rm Porous materials; granular materials; 82.80.Ej X-ray, Mössbauer and other γ-ray spectroscopic analysis methods

## Background

**Figure 1:**
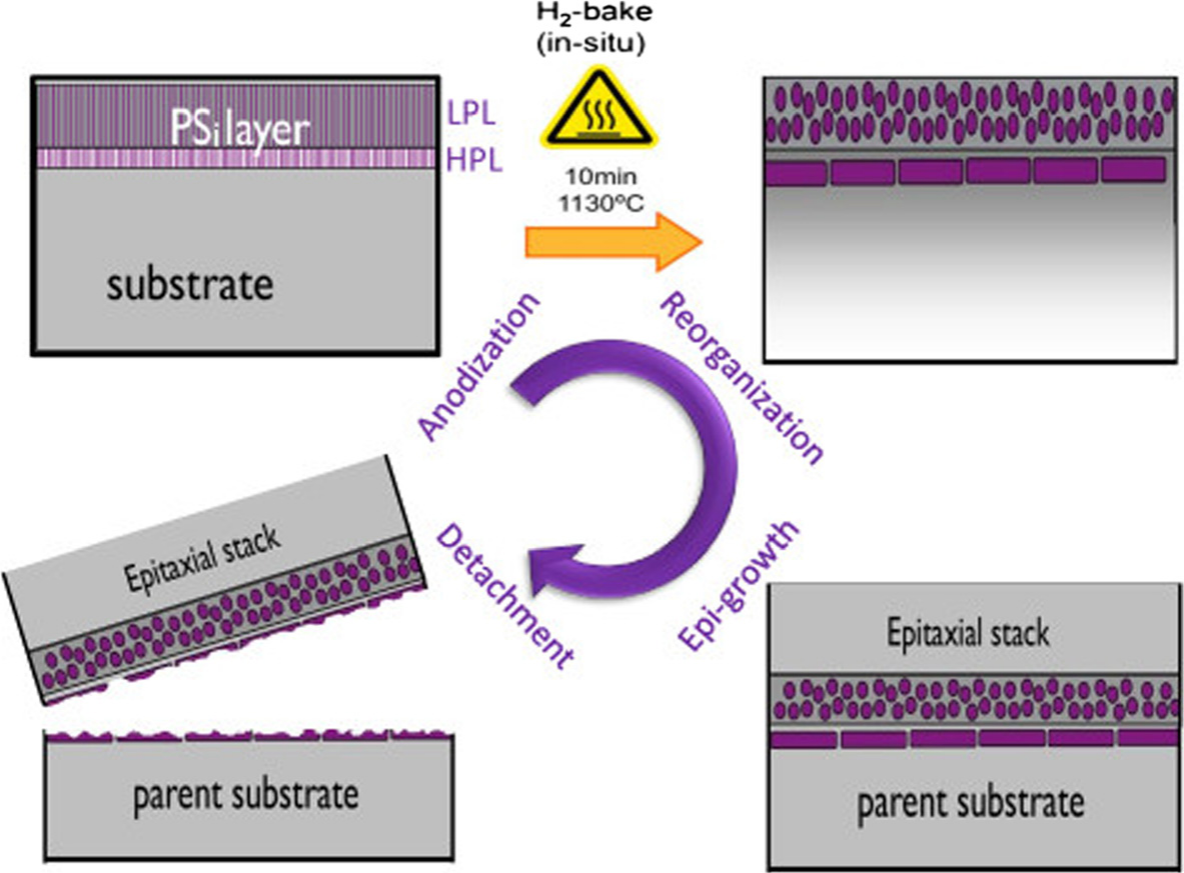
Schematic view of the PSi-based layer-transfer process.

Nowadays, about 30% of the cost of a wafer-based silicon solar cell is due to the silicon material itself. Thus, researchers are aiming at reducing the consumption of silicon while keeping the cell efficiency high. One of these attempts is employing a layer-transfer process (LTP) where an active silicon layer is epitaxially grown using chemical vapor deposition (CVD) on porous silicon (PSi), which acts as the detachment layer and as the epitaxy-seed layer [1,2]. Transferring the epitaxial layer (silicon “epi-foils”) to foreign low-cost substrates, while the parent substrate can be reused, would allow for cost-effective solar cells. In this PSi-based LTP, a double-PSi layer, with a low-porosity layer (LPL) on top of a high-porosity layer (HPL) is formed on a monocrystalline wafer by electrochemical etching and is sintered in hydrogen ambient, as schematically illustrated by the process flow in Figure 1. The HPL reorganizes into an extended void which serves as mechanically weak layer (i.e., the detachment layer) allowing the separation of the epi-foil from the parent substrate after the epitaxial growth. In addition, the LPL acts as “the seed layer” for the homo-epitaxial growth in which the columnar pores reorganize into large cavities while closing and smoothening the surface of the substrate. In most LTP schemes, a foreign substrate is used to provide mechanical support to the epi-foils during and after detachment. The efficiency of the silicon solar cells is influenced by the quality of the epitaxial growth, which is determined by the quality of the seed layer template. The PSi layer can influence the quality of the epitaxial growth in many ways. Firstly, since the LPL surface is the template where the epitaxial growth starts, the morphology and the topography of the LPL will affect the epitaxial growth process. Secondly, the intrinsic stress present in the PSi layer causes strain during epitaxial growth or even during the cool down [3].

In particular, strain in the whole PSi stack and surface roughness of the LPL are two major factors that drastically influence the epitaxial growth because of their role in the creation of dislocations, stacking faults, and other crystalline defects during epitaxy. Firstly, the lattice parameter of the as-etched PSi is in fact slightly larger than that of Si. This mismatch results in a contraction of the crystal planes of PSi in order to attain the same interatomic spacing as the Si substrate. As a result, a slight out-of-plane expansion (or tensile strain) is produced in PSi [4]. This tensile strain increases with porosity and the mean pore radius [4]. X-ray diffraction (XRD), especially in the high-resolution configuration (HR-XRD) was employed to detect this strain. Early attempts to determine strain in PSi were carried out by Barla et al. using a double-crystal diffractometer with a single silicon monochromator [4]. Afterward strain characterization using HR-XRD based on a four-reflection Ge monochromator becomes the most common [5]. Secondly, considering surface roughness, it is well known that crystalline defects inside epitaxial layers increase with the surface roughness of the seed layer.

Both strain and roughness of the seed layer can be reduced by optimizing the PSi stack, which is by fine-tuning the layer thicknesses and annealing time before epitaxy. Previously, Sivaramakrishnan Radhakrishnan et al. used micro-Raman measurements on annealed PSi to show that tuning the porosity and thickness of the LPL can result in a smoother seed surface with a lower residual stress distribution in the PSi stack. Subsequently, this leads to a lower epi-foil defect density [3]. Alternatively, Martini et al. used high-resolution profilometry (HRP) measurements to show how to obtain smoother annealed seed layers, which in turn result in a higher epitaxy quality [6]. In addition, G. Lamedica et. al showed that lattice deformation of both PSi layer and Si epitaxial layers grown on PSi strongly depends on the PSi porosity. They also showed that the epilayers grown on double-porosity layers have a high quality compared to films grown on n^+^-type single crystal Si substrates [7].

In this work, we present a fundamental investigation for the effect of the thickness of PSi and of its sintering time on strain and surface roughness. Strain is monitored on mono- and double-PSi layers by HR-XRD and surface roughness by HRP. In the first part, we study the impact of PSi thickness and present a model to support our observation of the strain reduction with a thicker LPL in a double layer of PSi. In the second part, we underline the change in strain type upon annealing, and then emphasize the antagonistic impacts of annealing time on strain and surface roughness. We correlate the strain reduction of the whole PSi stack to the HPL morphology, which is with the disappearance of the interconnections. The final aim is to provide guidelines for the optimum conditions of the epitaxial growth on a PSi stack, that is a smoother and minimum-strained PSi stack.

## Methods

**Figure 2:**
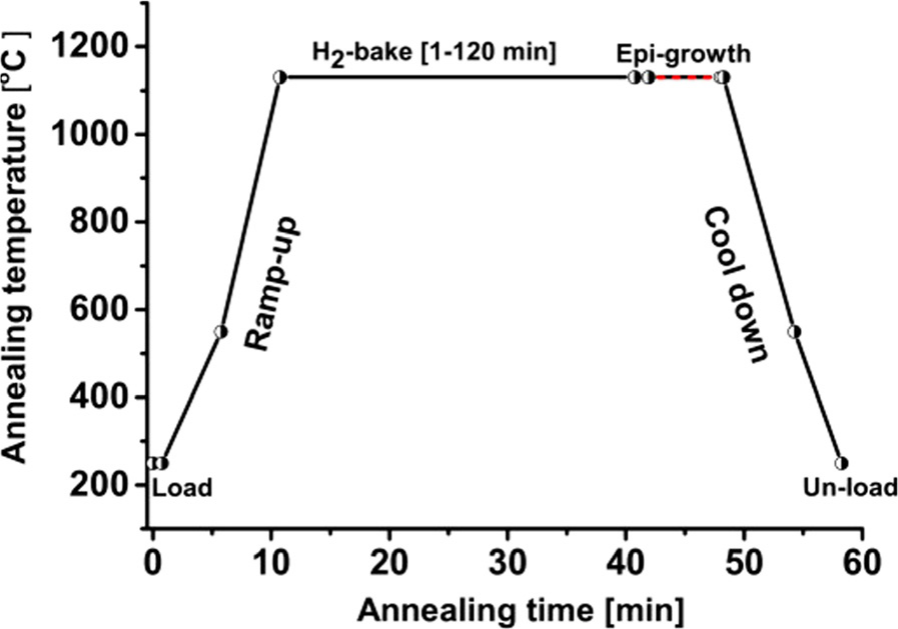
**Schematic view of the temperature profile.** The solid line represents the typical profile of the annealing and the dotted line represents the additional time for the epitaxial growth.

PSi was formed by electrochemical etching of 10 × 10 cm^2^ p-type mirror-polished Cz silicon wafers with boron doping level 10^19^ cm^−3^, under anodic bias and using an electrolyte of HF/ethanol mixture. A Teflon cell, with a platinum cathode and the silicon substrate as the anode, was used. PSi mono- and double-layer stacks were etched in galvanostatic mode at various current densities, as shown in Table 1. The porosity of the various layers was determined by the gravimetric method, using a cross-sectional scanning electron microscopy (SEM) view to determine the layer thickness. Afterward, the samples were annealed in a commercial epitaxial reactor (ASM Epsilon 2000, Conquer Industries, Inc, Union City, CA, USA), a single-wafer atmospheric-pressure chemical vapor deposition system (APCVD), at 1,130°C in 1 atm of H_2_ ambient for various durations between 1 and 120 min. The reorganization rate of the samples was fully reproducible for the samples in the same batches, i.e., annealed at the same moment of time. However, this reproducibility is affected for samples from different batches, probably due to the ageing of the epi-reactor. In this article, all samples shown on the same figure were loaded in the same batches, except for one figure that will be specified. A schematic representation of the temperature profile inside the reactor is shown in Figure 2, where the solid line shows the typical temperature profile for PSi annealing. The dashed line shows the additional time of epitaxial growth, which was not performed in the present work in order to maximize the XRD signal from the PSi stacks. Lattice strain was estimated by X-ray diffraction through symmetric (004) reciprocal lattice point with high-resolution Omega-2theta scans, which were performed in Bede Metrix-L (Bede Scientific, Durham, England). The source was monochromatic CuKα1 radiation (*λ* = 1.54056 Å) collimated by a four-reflection Ge monochromator with a beam size of 1 cm. In addition, a Gaussian fitting for the PSi peak was performed to some XRD profiles. The surface roughness of the sintered PSi stacks was investigated by a stylus-based HRP measurement using a HRP-200 (distributed by KLA Tencor, Milpitas, CA, USA), with a resolution of 5 nm. The RMS roughness values given are the average of three measurement points. Two types of scans were used, firstly, over areas of 20 × 20 μm^2^ with 21 lines spaced of 1 μm and, secondly, an area of 100 × 100 μm^2^ with the same pitch. The PSi layer’s morphology was examined by SEM to determine the thickness of the PSi layers, to capture the evolution of the pillars in the HPL and to monitor the bigger pores at the top surface of the PSi seed layers.

**Table 1:** Etching conditions of all the investigated samples for the three different studies of the impact of layer thickness, annealing time, and pillars on strain and surface roughness

**Parameters**	**Impact of thickness**			**Impact of annealing time**			**Pillars evolution**			
PSi stack	Monolayer	Double layer		Double layer			Double layer			
		LPL	HPL	LPL		HPL	LPL	HPL-1	STDHPL	HPL-2
Current density [mA/cm^2^]	1.4	1.4	73	1.4		73	1.4	50	73	97
Porosity [%]	30 ± 5	30 ± 5	55 ± 5	30 ± 5		55 ± 5	30 ± 5	ND	55 ± 5	ND
Etching time [s]/thickness [nm]	150/350	30% ± 5%	6/300	(I)	300/750	6/300	300/750	8/300	6/300	4/300
	300/750	50/150								
	600/1300	150/350								
	900/1700	300/750								
		450/900		(II)	600/1300	6/300				
		600/1300								
		900/1700								
		1200/2000								

## Results and discussions

### Effect of PSi layer thickness on strain and surface roughness

#### The case of PSi monolayers

**Figure 3:**
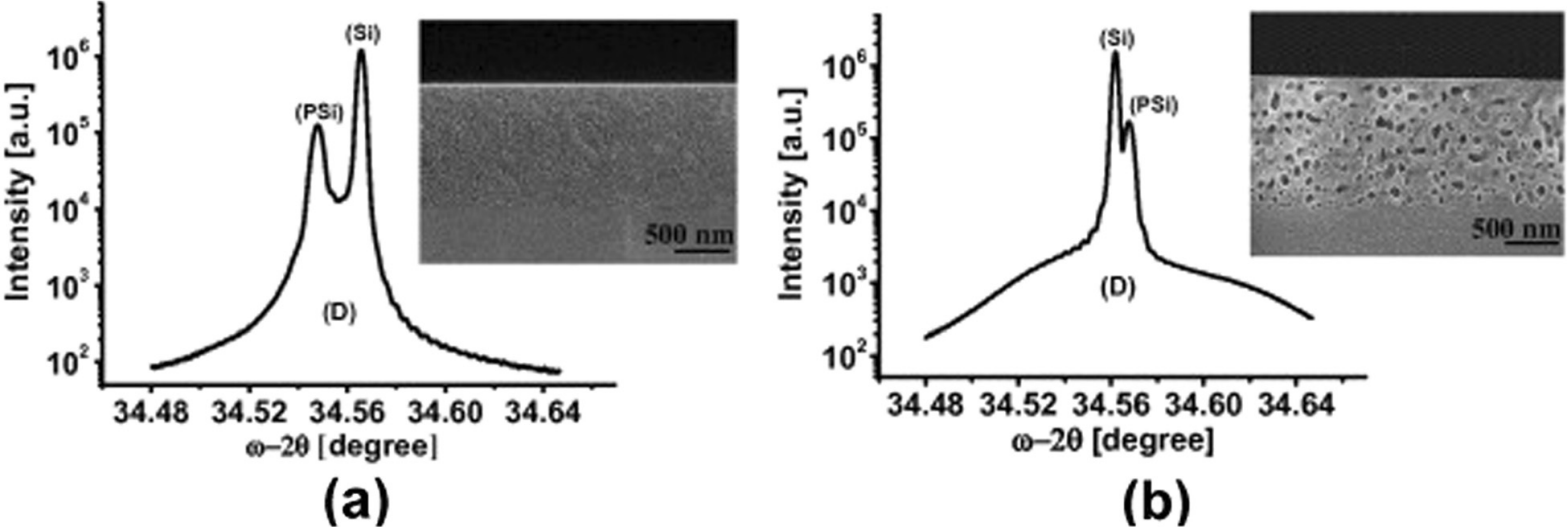
**XRD profiles of the as-etched and the annealed, 1,300-nm-thick, low-porosity monolayer of PSi.** XRD profiles combined with the cross-sectional SEM image of the as-etched **(a)** and annealed **(b)** monolayer of PSi, 1300-nm-thick, displaying two clear peaks corresponding to the Si substrate and the PSi layer, on top of a broad hump (D). Upon annealing, the PSi peak shifts from lower to higher angle relative to the Si-peak, indicating a change in the out-of-plane strain from tensile to compressive.

To investigate the effect of the thickness of the PSi stack (monolayer and double layers), on the strain and surface roughness, several PSi layers were prepared with different thicknesses and porosities as summarized in Table 1 (column “Impact of thickness”). Figure 3 shows the XRD profiles of the as-etched and the annealed, 1,300-nm-thick, low-porosity monolayer of PSi of about 30% ± 5% of porosity. That XRD profile (plotted on a semi-logarithmic scale) is typical for a PSi layer attached to a Si substrate showing two characteristic peaks (see Figure 3). The higher intensity peak corresponds to the monocrystalline silicon substrate while the lower intensity peak is due to the PSi layer. Upon annealing, the PSi peak shifts from lower to higher angle relative to the Si-peak, indicating a change in the type of the out-of-plane strain (i.e., tensile to compressive). A broad hump (D), which is reported also by Bensaid et al. [8], is observed below the two narrow peaks. This is due to the diffuse scattering caused by the presence nanometric structure of silicon crystallites. The relative expansion or contraction *Δa*/*a* in the PSi lattice structure with respect to the silicon substrate along the (001) direction perpendicular to the sample surface is directly proportional to the angular splitting *Δθ*_*B*_ between the two XRD spectrum peaks [9]: *Δa*/*a* = −*Δθ*_*B*_ cot *θ*_*B*_ where *θ*_*B*_ is the Bragg’s angle.

**Figure 4:**
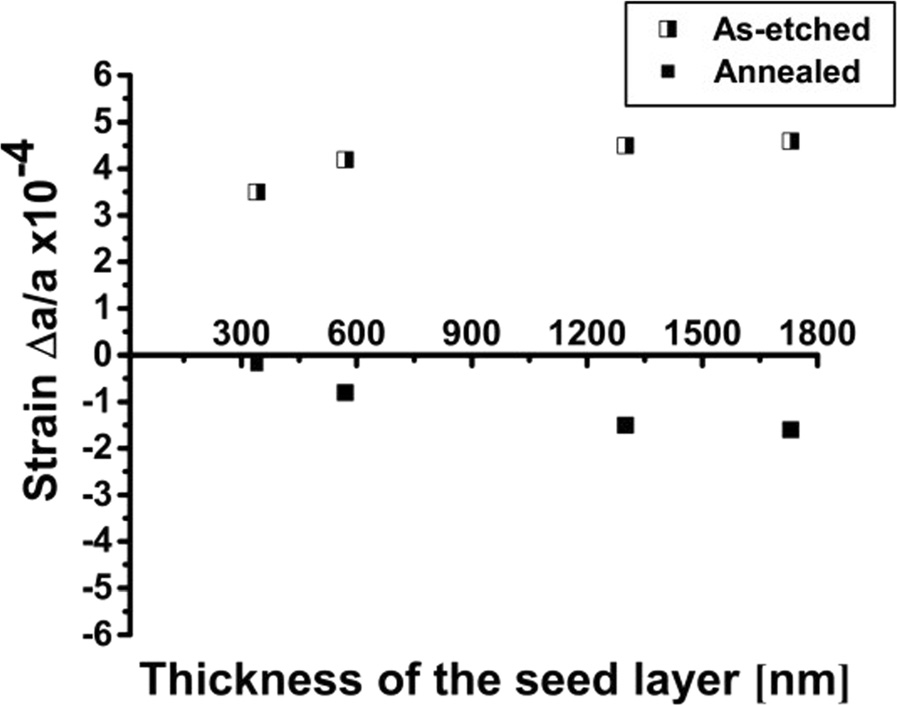
**Comparison between the out-of-plane strain values in as-etched (semi-solid) and annealed (solid) monolayers of PSi.** Both showing an increasing strain with thickness, but with opposite signs.

The PSi peak is at a lower angle relative to the Si reference peak. This is the case for all the as-etched samples but with different angular splitting *Δθ*_*B*_ between the two peaks. This splitting between the two peaks increases as the thickness of the monolayer of PSi increases from 350 to 1,700 nm. This indicates an increase in the expansion of the PSi lattice in the normal direction to the Si-substrate, implying a ~26% incremental increase in the out-of-plane tensile strain from 3.5 × 10^−4^ to 4.6 × 10^−4^, as depicted by the semi-solid squares in Figure 4.

A similar set of samples with PSi monolayers were annealed for 10 min in H_2_-ambient at 1,130°C. As shown in Figure 4, the strain increases with increasing thickness of the annealed PSi monolayer. This trend is identical to that of the as-etched case, but with an opposite sign, i.e., compressive strain. In fact, the increase in the thickness of the annealed monolayer of PSi from 350 to 1,700 nm resulted in ~88% incremental increase in the out-of-plane strain from 0.2 × 10^−4^ to 1.6 × 10^−4^, as depicted in Figure 4 by the solid squares.

Two effects are thus simultaneously occurring for the PSi upon annealing, strain conversion from tensile to compressive and strain reduction. It is well known that the PSi lattice mismatch parameter is very sensitive to the chemical state of PSi internal surface [10,11]. The as-etched sample contains a high density of adsorbed hydrogen on its pore walls, which causes in-plane compressive stress on the pore side walls. That stress leads to out-of-plane expansion of the PSi lattice, resulting in the monitored out-of-plane tensile strain [10]. Likewise, desorption of hydrogen could be the main source of strain conversion. As proposed by Sugiyama et al., as the sample is annealed, most of this hydrogen is desorbed. This desorption leads to a considerable reduction in the in-plane compressive stress, leading to the relaxation of the lattice expansion in the in-plane direction and, conversely, to an out-of-plane compressive strain. Moreover, according to Chelyadinsky et al. [11], a disordered thin film of amorphous silicon, which conformably covers the pore wall, is also present and a main reason for the lattice deformation. In their work, they showed that the recrystallization of this amorphous silicon film, in addition to the gas desorption in the higher temperature of vacuum annealing at 800°C, would lead to the relaxation of the PSi lattice parameter to the value of monocrystalline Si [11]. However, the measurements in [10,11] were performed on samples annealed in vacuum, while our case is in H_2_ ambient, and we would thus expect here some H-termination to the pore side walls during cooling down below the desorption temperature of Si-H_*x*_ bonds. We can speculate that during the cooling down, the coefficient of thermal expansion (CTE) of PSi is higher than that of Si, which leads to a faster in-plane contraction of the PSi layer compared to bulk Si. Bulk Si will block the in-plane contraction of PSi which, in turn, leads to in-plane expansion and out-of-plane contraction of the PSi layer. In this way, the strain becomes compressive rather than tensile. A further investigation will study the point of strain conversion and the H-termination during cooling down with Fourier-transform infrared spectroscopy in a future work. To understand the strain reduction upon annealing, one should recall that pore size, pore distribution, and porosity change upon annealing, as illustrated in the SEM insets of Figure 3. Upon annealing, the total PSi internal surface area reduces [9], which leads to a reduction in the areal density of Si-H bonds on the pore walls. This produces a lower in-plane compressive stress on the side walls and, in turn, a lower out-of-plane expansion strain is present in the smaller pore area annealed porous layer than in the larger pore area as-etched porous layer.

**Figure 5:**
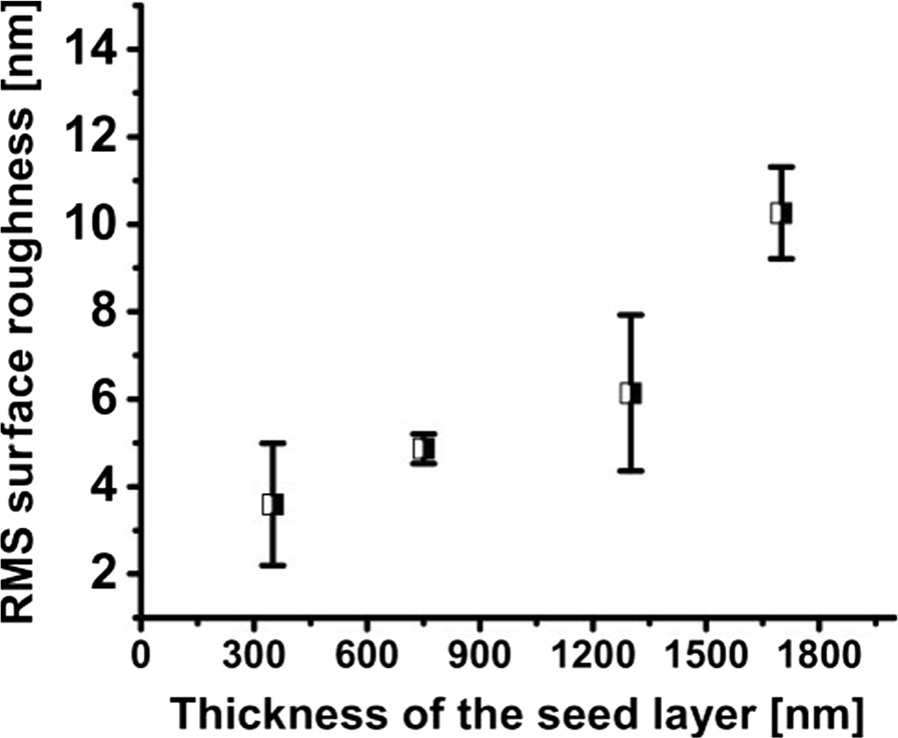
**RMS values for surface roughness of annealed monolayers of PSi samples with different thicknesses 350, 750, 1,300 and 1,700 nm.** The roughness increases as the thickness of the LPL increases.

After the out-of-plane strain, the surface roughness of the annealed PSi monolayers was measured and analyzed using HRP. Figure 5 shows that the surface roughness of the seed layer increases with its thickness, as also observed in [3] and [6]. This result may be explained in light of previous observations that thick PSi layers tend to have less aligned and larger pores at the top which, in turn, results in a rougher seed surface. An epitaxial growth template with a rough surface is likely to generate crystal defects in the epitaxial layer.

From the evolution of strain and roughness with layer thickness as observed with these low-porosity monolayers, a direct guideline would be to grow layers that are as thin as possible, in order to minimize both parameters. However, detachable epitaxial foils require formation of porous stacks with a double layer, with a LPL on top of a HPL. The evolution of strain in the double-porosity layers is investigated in the next section.

#### The case of PSi double layers

**Figure 6:**
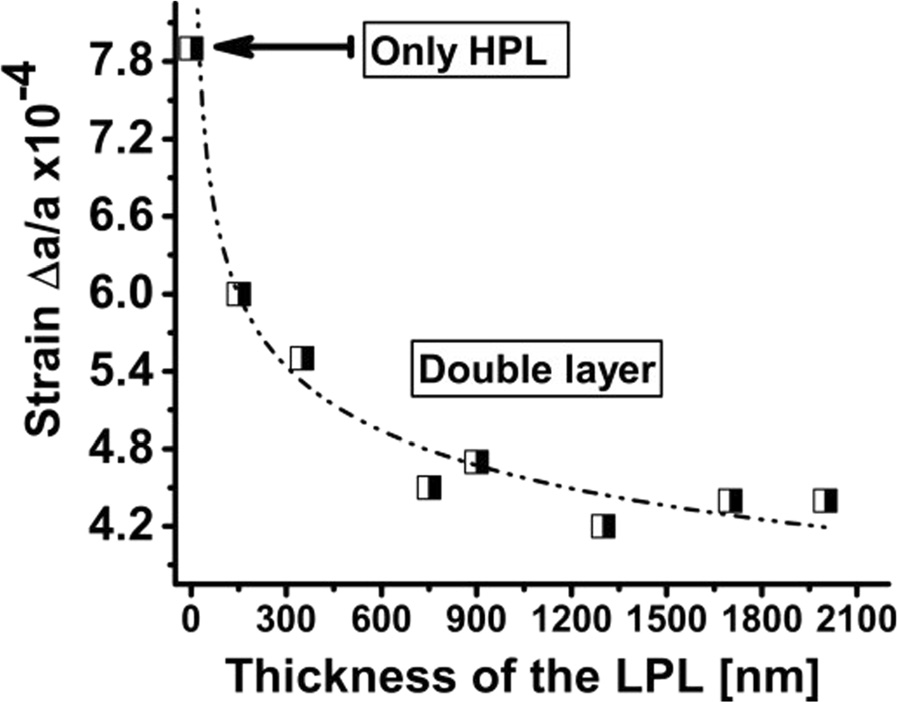
**Out-of-plane tensile strain values of the as-etched double layer of PSi.** Strain decreases and saturates as the LPL thickness increases, the dashed line is a trend for the eye. The maximum strain value corresponds to the high-porosity layer without any low-porosity on top (monolayer).

The evolution of out-plane strain in double layers was investigated by adding a high-porosity layer under the low-porosity layers. In particular, the thickness of the LPL was varied as in the previous section, while the HPL, with a porosity of 55% ± 5%, was kept constant, as detailed in Table 1 (column “Impact of thickness”). Similarly to the as-etched PSi monolayers, the strains in as-etched double layers were tensile, as illustrated in Figure 6. However, contrarily to the monolayers, we can observe that, unexpectedly, the total out-of-plane strain decreases with the thickness of the LPL and saturates.

The opposite behaviors of the strain in mono- and double-PSi stacks may be explained by taking into account the interaction between the HPL and the LPL. We are in presence of a LPL with lower-stressed pores (small size pores) on top of considerably higher-stressed pores (larger size) in the HPL [4]. The lower-stressed pores of the LPL will help the relaxation of the higher-stressed pores of the HPL through their interface. In the case of a thinner LPL, only a small force is exerted on the top of the HPL, leading to a minimal relaxation force of strain in the HPL pores. When the thickness of the LPL is increased, a higher force is exerted on the HPL, helping its pores to relieve more stress. Similarly, a HPL without any LPL on top results in the highest strain value, as illustrated experimentally in Figure 6. This shows that the main source of strain in a double layer of PSi is the strain which is coming from the HPL and that the LPL releases strain from this stack. Nevertheless, this model does not directly explain the asymptotic behavior of the strain as the LPL thickness increases.

To conclude, in case of double layer of PSi, a thicker LPL should be preferred for growing lower-strained stacks, and the interaction between the various stack components should be taken into account.

### Effect of annealing time on strain and surface roughness

**Figure 7:**
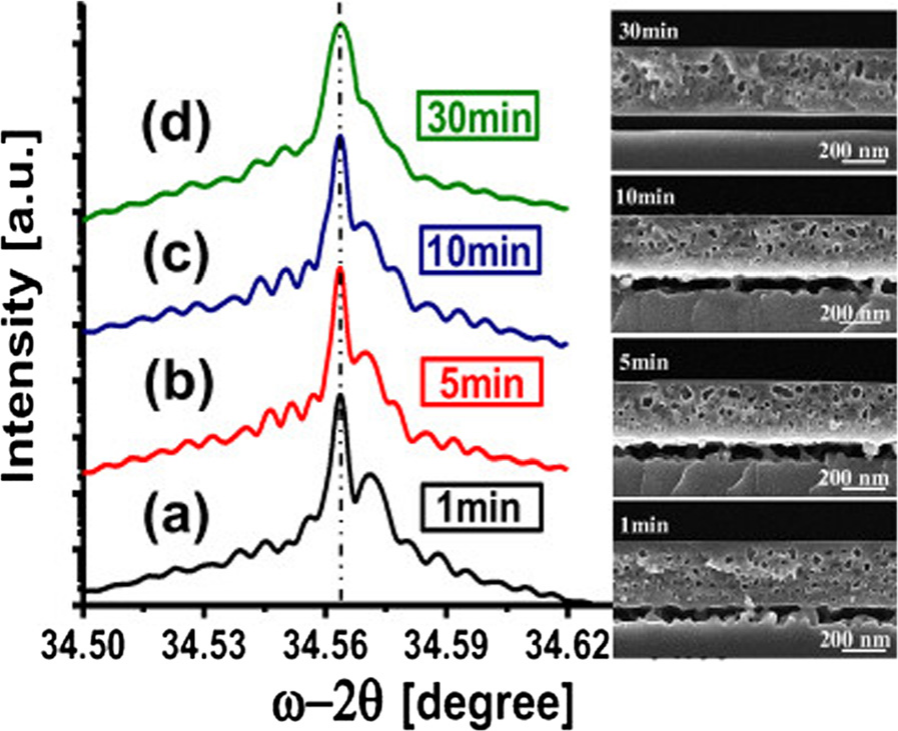
**XRD profiles of annealed double layers of PSi with cross-sectional SEM images of different annealing times (1, 5, 10 and 30 min).** The PSi-peak shift toward the Si-peak suggests a decrease of strain with annealing time that may be correlated with the disappearance of pillars in the HPL.

**Figure 8:**
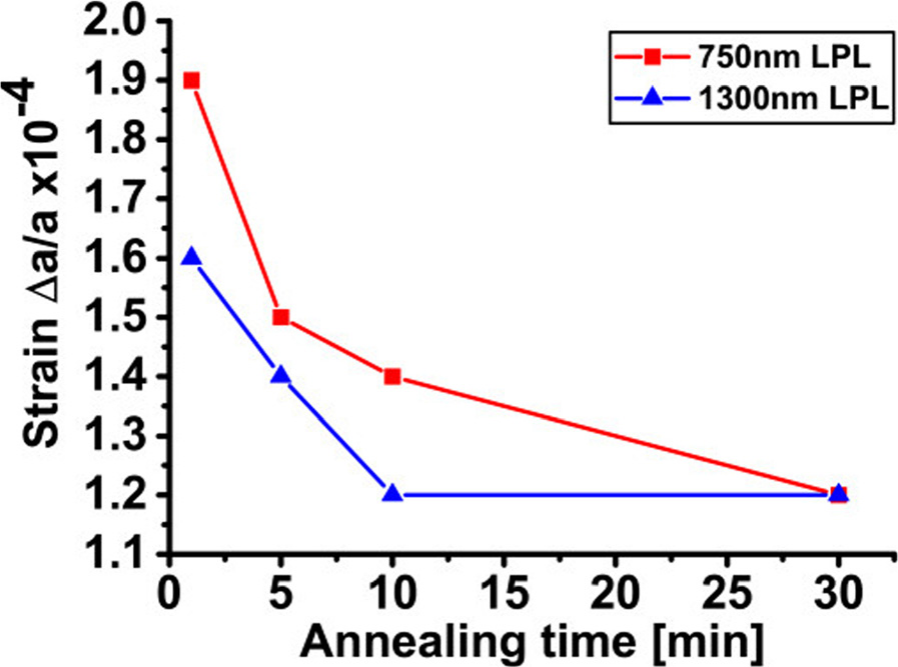
**The out-of-plane compressive strain values of the annealed double layer of PSi with 750- and 1,300-nm-thick LPL.** Strain is released gradually from the layers as the annealing time increases. Similarly to the as-etched samples, a thicker LPL leads to a lower-strained stack, but strains equalize for longer annealing times.

After monitoring as-etched double layers, the effect of annealing time on the strain and surface roughness was investigated on stacks with a fixed LPL and HPL, as listed in Table 1 (column “Impact of annealing time”). Figure 7 shows XRD profiles of the annealed double layer of PSi. Similarly to the case of PSi monolayers, the strain switches from tensile to compressive after annealing. Furthermore, the angular splitting of the XRD peaks decreases as the annealing time of the double layer of PSi increases over the investigated range. This indicates a ~37% incremental decrease in the out-of-plane compressive strain from 1.9 × 10^−4^ to 1.2 × 10^−4^, as shown in Figure 8. Finally, a thicker-LPL stack shows a lower strain than a thinner-LPL stack, as shown in Figure 8 with two LPL of 750- and 1,300-nm thickness.

**Figure 9:**
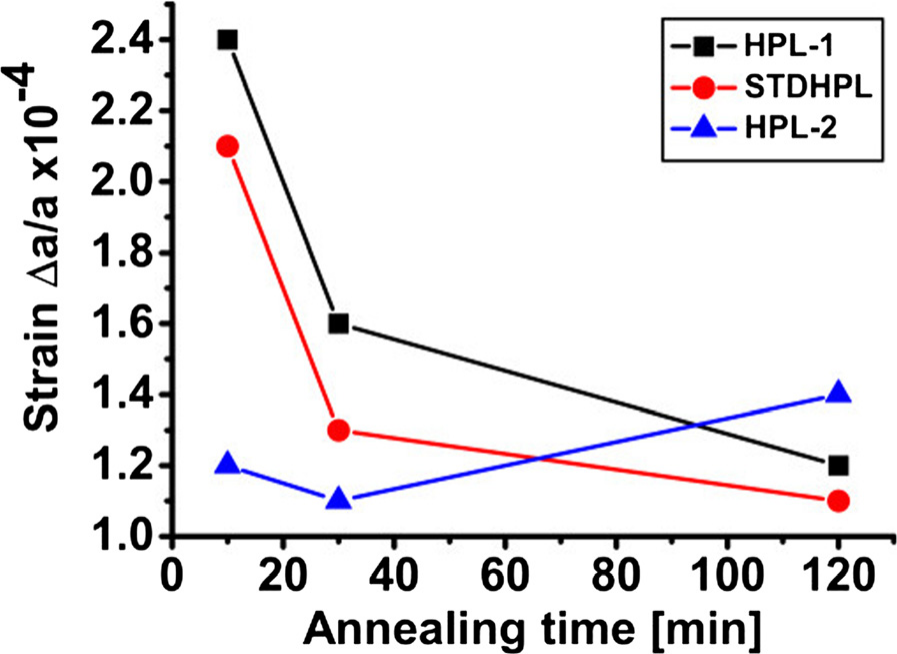
**The out-of-plane compressive strain values of the annealed double layer of PSi with different HPL porosities.** Strain is released from the PSi stack with annealing time, which is correlated to the progressive reduction of pillars connecting the PSi to the bulk Si.

**Figure 10:**
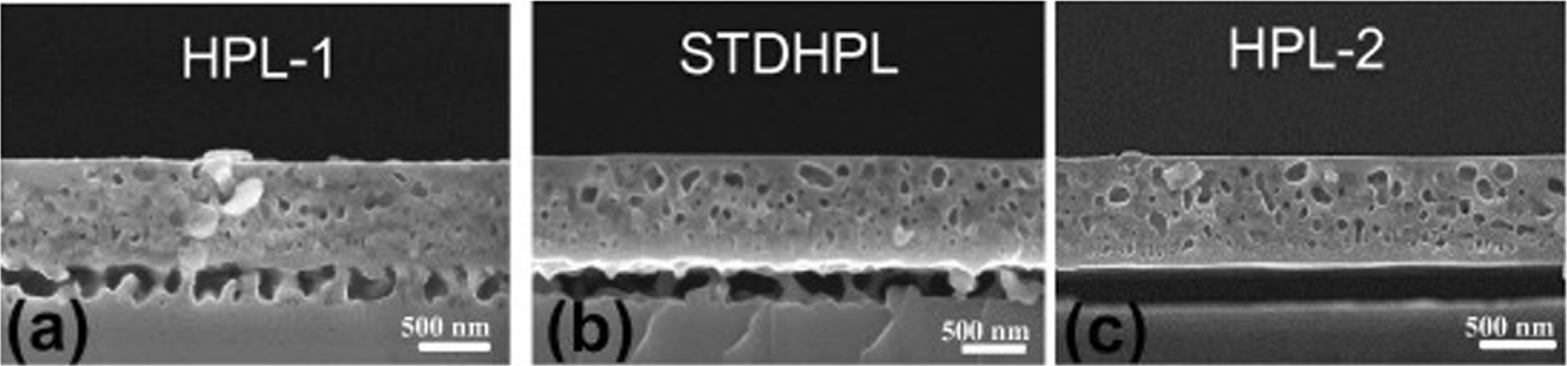
**Cross-sectional SEM images of double layer PSi annealed for 10 min with identical LPL but with different HPL porosities. (a)** Lower porosity (HPL-1), **(b)** standard porosity (STDHPL), and **(c)** high porosity (HPL-2), showing the gradual disappearance of the inter-connection pillars in the HPL with increasing porosity.

To understand the impact of annealing time on strain of the double-PSi layer, one has to recall that the strain in the HPL seems to dominate that of the entire stack and to consider the morphological evolution of the stack. While the LPL, as in the monolayer case, transforms into spherical voids with lower surface area and facets, the HPL becomes almost 100% porous, with a few silicon “pillars” connecting the LPL to the Si bulk (see SEM images of Figure 7). The gradual disappearance of these pillars by increasing the annealing time can be expected to result in a relaxation of the whole stack and a decrease in strain, since the disappearance of connections between the LPL and the bulk releases the two mismatched lattices at the origin of strain. To provide support for this hypothesis of the role of the HPL’s pillars in releasing the strain of the entire stack, samples were prepared with the same LPL but different HPL porosities, as detailed in Table 1 (column “Pillars evolution”). Samples with lower (HPL-1), standard (STDHPL), and higher (HPL-2) porosity HPL were prepared. The etching time during the HPL formation was adjusted to ensure that all samples keep the same thickness of 300 nm. The annealing temperature was kept constant while the annealing time was varied (10, 30, and 120 min.). Figure 9 shows the out-of-plane compressive strain for the annealed double layer of PSi at different HPL porosities. The strain of the whole PSi stack tends to decrease with annealing time, as previously observed, except for the HPL-2 annealed for longer 120 min. That sample however, because of its very low pillar density, showed a tendency for flaking when handled, which made the measurement difficult. Besides, it is possible that the foil may have locally collapsed on the bulk parent wafer, that behavior being frequent for such unstable stacks. Finally, for a given annealing time, the strain decreases with increasing the porosity of the HPL, e.g., with lowering the density and/or the number of the pillars in the HPL. The cross-sectional SEM monographs in Figure 10 depict the disappearance of the pillars in the HPL-2, compared to STDHPL and HPL-1.One notice is to be added on the discrepancy between the strain values of the two samples with a LPL 750-nm thick annealed for 10 min in Figures 8 and 9. We believe this difference could be attributed to the different reorganization rate, which is dependent on the ageing of the tube of the Epi-reactor (as mentioned in the “Methods”), since the two samples were loaded inside the tube at different moments in time. In fact, this reorganization rate affects the evolution of the pore shape and of the pillar “inter-connections” between the Si-substrate and the seed layer and, hence, the strain values. The sample in Figure 8 has a strain value lower than its counterpart in Figure 9. This is seemingly a result of the slower rate of reorganization, which is indicated by the slightly larger number of pillars in the SEM images.

**Figure 11:**
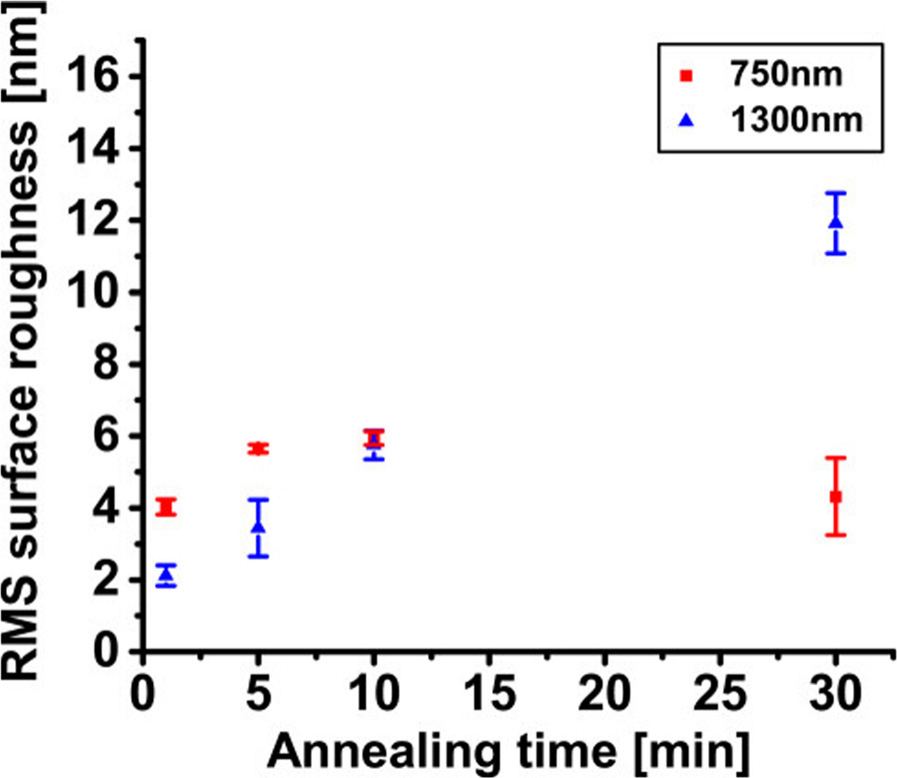
**RMS values of the LPL surfaces of the annealed PSi double layer.** RMS values of surface roughness of the annealed double layer of PSi, with 750- and 1,300-nm thick LPL, as a function of annealing time (1, 5, 10 and 30 min). The roughness increases slightly from 1 to 10 min and becomes unstable for longer times.

**Figure 12:**
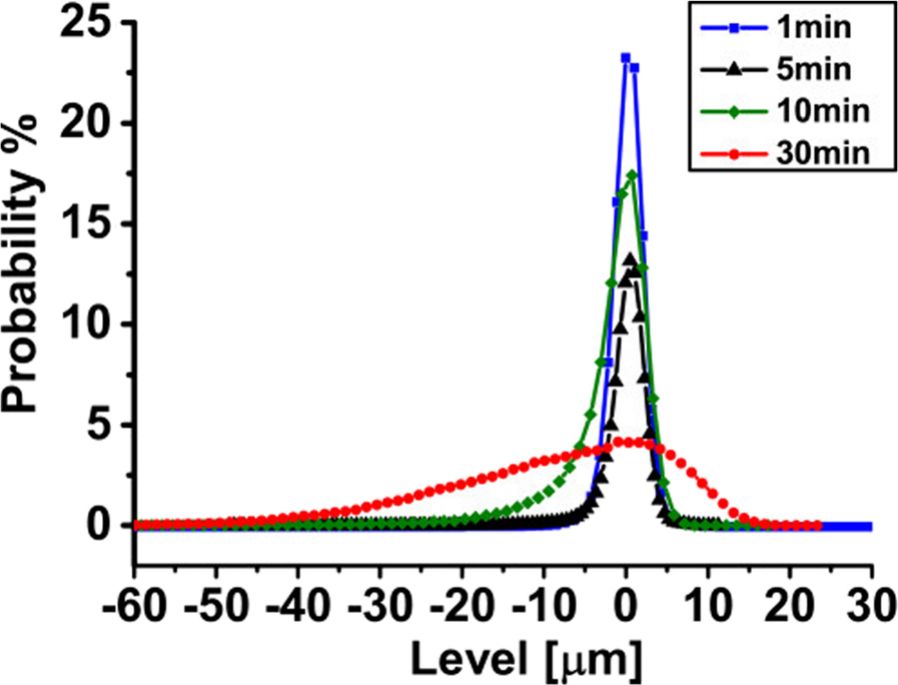
**Derivative of BAC of PSi double layers with 1,300-nm-thick LPL annealed for 1, 5, 10 and 30 min.** The asymmetries toward the negative *x*-axis increase as the annealing time increases. This shows that the density of holes in the seed layer increases for long annealing times.

To conclude on the impact of annealing time on the PSi stack, the surface roughness of the seed layer was also analyzed for two double porous silicon layers with LPL of 750- and 1,300-nm thickness. Figure 11 shows the RMS values of the LPL surfaces which vary slightly, and then show a sudden increase at longer annealing time for the thicker-LPL double stack. This observation may be understood in light of the fact that a longer annealing time results in formation of larger pores, which coarsen at the very top surface of the seed. Accordingly, large valleys (holes) may appear sporadically on the surface, which results in a rougher surface. Figure 12 shows the derivative of the bearing area curve (BAC) for the larger scanned area of the thicker-LPL sample. It was observed that there is no significant change in RMS roughness values between smaller (20 × 20 μm^2^) and larger (100 × 100 μm^2^) scanned areas. However, the increase of the non-symmetries of the graphs upon longer annealing times indicates an increase in the probability of the presence of holes. As the annealing time increases, the asymmetry of the curves is pushed toward the negative *x*-axis, which indicates the increased density of holes - as opposed to bumps - in the seed layer upon longer annealing.

To conclude, we can see that the evolution of strain and roughness with layer thickness and annealing time go in opposite directions. While reduction of strain calls for thicker double-PSi stacks and longer annealing times, roughness calls for thinner double-PSi stacks and shorter annealing times. Finding a trade-off between the two effects is therefore necessary.

## Conclusions

In this work, we studied the impact of two factors on the quality of highly boron doped PSi double layers as epitaxy seed layers: strain and surface roughness. We investigated mainly two parameters which have an impact on these factors, acting either on the etching process or on the subsequent thermal treatment, seed layer thickness and annealing time. This work aims at tuning these parameters to minimize strain and surface roughness of the PSi stack which, in turn, affects the epitaxial growth and thus the presence of crystalline defects in the epitaxial foils.

For monolayers of PSi, our results reveal that strain and surface roughness decrease by decreasing the thickness of the layer. A similar behavior was observed for as-etched monolayers and for annealed monolayers, but with higher absolute values and opposite sign. As expected, annealing has an effect of strain relief related to the morphological changes implied by the sintering. Moreover, surface roughness also increased with layer thickness. This was attributed to the bigger pore formation at the top surfaces of thicker PSi layers. Therefore, all these results suggest that, both in terms of strain and surface roughness, thinner PSi layers would be better and highly preferred for high-quality epitaxial growth. However, for forming detachable epitaxial foils, a HPL is to be included below the seed layer. And, unexpectedly, strain decreased and saturated, by increasing the thickness of the LPL. We explained this by proposing to consider the interaction between the strain in the HPL and the LPL at their interface and that the dominating source of strain in the double layer of PSi is coming from the HPL.

Also, our results reveal that strain is released gradually from double layers of PSi by longer annealing times. This was attributed to the disappearing of the inter-connections between the porous seed layer and the Si substrate. The exposure to longer annealing times of the double layer of PSi results in fact in a lower density of pillars that, in turn, results in a lower out-of-plane compressive strain. This interpretation was supported by measurements on samples with higher and lower porosity HPL, with higher and lower density of pillars, respectively. However, if longer annealing times result in lower strain, they may conversely result in a significant increase in surface roughness, due to the occasional opening of pores at the very top surface over time.

Finally, for a multi-layer stack of PSi, which is a must to combine ease of foil detachment and good crystalline quality, thicker LPL and longer annealing times help in reducing strain but produce a rougher surfaces. A trade-off between these effects, of lower-strained stack and rougher seed, is required for finding the optimum condition for a better seed template for higher quality epitaxial growth. Further work will therefore focus on investigating directly the crystalline quality of epi-foils grown on seeds of various annealing times and thicknesses, in order to identify the dominating effects.

## Abbreviations

PSi: porous silicon; Si: silicon; LTP: layer-transfer process; HPL: high-porosity layer; LPL: low-porosity layer; CVD: chemical vapor deposition; APCVD: atmospheric-pressure chemical vapor deposition; XRD: X-ray diffraction; HR-XRD: high-resolution X-ray diffraction; HRP: high-resolution profilometry; RMS: root mean square; SEM: scanning electron microscopy; CTE: coefficient of thermal conductivity; BAC: bearing area curve.

## Competing interests

The authors declare that they have no competing interests.

## Authors’ contributions

For the technical issues, MK performed XRD, HRP and SEM and wrote the manuscript. RM performed HRP and SEM. HSR performed SEM. KVN performed annealing in the Epi-reactor. VD supervised and designed the work and reviewed and proofread the manuscript. JP is the promoter. IV, WR and JP contributed to the discussions. All authors read and approved the final manuscript.

## Authors' information

MK is a joint PhD student at Alexandria University, Egypt, and KU Leuven, Belgium. RM is a PhD student at KU Leuven. HSR is a researcher in Silicon Photovoltaics at IMEC, Belgium. VD is a research engineer in Silicon Photovoltaics at IMEC. KVN is a research engineer in Silicon Photovoltaics at IMEC. WR is an Associated professor at Physics Department at Alexandria University, Egypt. IG is the manager of Silicon Photovoltaics at IMEC, Belgium. JP is a professor at ESAT Department of KU Leuven and the photovoltaics program director at IMEC, Belgium.
